# Intravital two-photon microscopy of the native mouse thymus

**DOI:** 10.1371/journal.pone.0307962

**Published:** 2024-08-01

**Authors:** Negar Seyedhassantehrani, Christian S. Burns, Ruth Verrinder, Victoria Okafor, Nastaran Abbasizadeh, Joel A. Spencer

**Affiliations:** 1 Quantitative and Systems Biology Graduate Program, University of California Merced, Merced, California, United States of America; 2 NSF-CREST Center for Cellular and Biomolecular Machines, University of California Merced, Merced, California, United States of America; 3 Department of Bioengineering, University of California Merced, Merced, California, United States of America; 4 Health Science Research Institute, University of California Merced, Merced, California, United States of America; Universite Paris-Saclay, FRANCE

## Abstract

The thymus, a key organ in the adaptive immune system, is sensitive to a variety of insults including cytotoxic preconditioning, which leads to atrophy, compression of the blood vascular system, and alterations in hemodynamics. Although the thymus has innate regenerative capabilities, the production of T cells relies on the trafficking of lymphoid progenitors from the bone marrow through the altered thymic blood vascular system. Our understanding of thymic blood vascular hemodynamics is limited due to technical challenges associated with accessing the native thymus in live mice. To overcome this challenge, we developed an intravital two-photon imaging method to visualize the native thymus *in vivo* and investigated functional changes to the vascular system following sublethal irradiation. We quantified blood flow velocity and shear rate in cortical blood vessels and identified a subtle but significant increase in vessel leakage and diameter ~24 hrs post-sublethal irradiation. *Ex vivo* whole organ imaging of optically cleared thymus lobes confirmed a disruption of the thymus vascular structure, resulting in an increase in blood vessel diameter and vessel area, and concurrent thymic atrophy. This novel two-photon intravital imaging method enables a new paradigm for directly investigating the thymic microenvironment *in vivo*.

## Introduction

The thymus is a primary lymphoid organ essential for T cell development, and it is a cornerstone of the adaptive immune system and overall human health [1, 2]. T cell development relies on the complex interaction between thymus stromal cells (e.g., thymic epithelial cells (TEC), blood vascular endothelial cells (EC), and fibroblasts) and thymocytes which themselves are dependent on the recruitment of *de novo* “seeding” early thymic progenitor cells (ETPs) from the bone marrow (BM) [3, 4]. The thymus is extremely sensitive to a range of acute and/or chronic insults, such as stress (corticosteroids), infection, sex hormones, and many cytoreductive treatments including chemotherapy, radiotherapy, and antibody therapy [5]. For example, the damage caused by cytoreductive treatments in patients undergoing hematopoietic cell transplantation (HCT) inhibits the capacity of the thymus to produce functional T cells contributing to increased morbidity and mortality [4, 6–9]. Endogenous thymus regeneration after total body irradiation depends on the engraftment of BM lymphoid progenitors as ETPs in the thymus. They mobilize into the bloodstream from the BM and traffic to the thymus through the blood vascular system and enter the thymus at the cortico-medullary junction [10–13]. ETPs then interact with thymic ECs and TECs to begin the differentiation process into distinct T-cell subsets [10, 14–19]. The thymus vasculature and ECs play critical roles as the highways of entry and a source of cell signaling, respectively, during the early stages of endogenous thymic regeneration [10, 13, 18].

It was previously reported that thymic ECs are radio-resistant and that the absolute number of ECs does not significantly decrease after sublethal total body irradiation (SL-TBI) [20]. Nevertheless, visualization of the thymic blood vessel network using light-sheet fluorescence microscopy 4 days after SL-TBI revealed significant changes to the thymus vasculature, including decreases in the total volume of the vessel network, total number of vessel segments, average vessel length, and vessel branching [20]. Notwithstanding the critical role of ECs in thymic regeneration, it remains unclear if changes to the vascular network occur earlier than 4 days and whether these changes alter thymic hemodynamics or other vascular functions.

To study functional changes to the vasculature, video-rate intravital microscopy with subcellular resolution is required, but direct visualization of the native thymus in live mice has been elusive and deemed impossible with two-photon microscopy [21]. The position of the thymus directly dorsal to the sternum and internal thoracic vein and cranial to the heart and lungs, introduces several logistical, mechanical, and health complications when attempting to image the thymus. Commonly used analysis methods such as flow cytometry, immunohistochemistry, and imaging of excised tissue have been applied to study the thymus in preclinical mouse models but these methods are limited to *ex vivo* analysis. *Ex vivo* culture systems [22, 23], including thymic slices [24–29] and whole organ imaging [30, 31], are very powerful systems for studying certain aspects of thymus biology such as thymocyte-stromal interactions [32, 33] and T cell development [27, 34], but these techniques lack blood flow and may not fully recapitulate the *in vivo* situation. Due to a lack of viable intravital imaging methods for the native thymus, researchers have relied on thymus transplantation to optically accessible sites including the kidney capsule [35–38], anterior chamber of the eye [39], and pinna of the mouse ear [40] for intravital microscopy, but transplantation alters the native vascular connections and exposes the tissue to an aberrant microenvironment [37, 41]. Although it has been reported that the transplanted thymus mirrors the endogenous thymus in architecture and vascularity, direct measurement of hemodynamic parameters in the native thymus is yet to be reported [41]. Teleost fish have been suggested as a viable alternative to mice models since the native thymus can be directly observed *in vivo* [42–45]. In mammals such as mice and humans, T cell development critically and nonredudantly depends on IL-7 and Foxn1, which is not the case in teleost fish, however [46, 47]. Therefore, tools to directly visualize the native mouse thymus are needed in order to study functional changes to the thymic vascular system after cytotoxic preconditioning in an immunologically similar model to humans.

Here, we developed a novel intravital imaging method using two-photon microscopy to visualize the native thymus in live mice without transplantation. This method utilizes a thoracotomy to access the thymus within the chest cavity [48], followed by the placement of a custom-designed adhesion stabilization holder to reduce vertical and lateral movement from the heart and lungs [49–54]. Using this method, we were able to directly investigate functional and anatomical changes to the thymic blood vascular network within 24 hrs after SL-TBI in live mice. Two-photon intravital imaging of the native thymus opens a new paradigm for studying thymus biology which was not previously possible.

## Materials and methods

### Experimental animals

Male and Female UBC-GFP (C57BL/6-Tg (UBC-GFP)30Scha/J) mice between the age of 8–12 weeks were used for intravital imaging experiments and wild-type (C57BL/6J) mice were used for *ex vivo* imaging experiments. Mice were housed under pathogen-free conditions in the University of California, Merced’s vivarium with autoclaved feed and water, and sterile microisolator cages. The University of California, Merced Institutional Animal Care and Use Committee (IACUC, A4561-01) gave written approval for all animal work. All mice were euthanized in accordance with IACUC-approved methods after imaging and before tissue collection.

### Animal irradiation

Mice received SL-TBI from a Precision X-Rad 320 at a dose of 4.5 Gy ~ 24hrs before intravital imaging or tissue extraction.

### Preparation of thymus for *ex vivo* whole organ imaging

Mice were anesthetized with isoflurane (induction 3%, maintenance 1.5%, 100% O_2_ at 1L/min). Fluorescent antibodies (anti-mouse Ly-6A (Sca-1) A647 Biolegend 108118, 6ug/100ul; anti-mouse CD144 (VE-cadherin) A647 Biolegend 138006, 5ug/100ul; and anti-mouse CD31 A647 Biolegend 102516, 5ug/100ul) were retro-orbitally injected to label blood vessels. 20 minutes after injection, animals were perfused intracardially with phosphate buffered saline (1X), followed by cold paraformaldehyde (4%, pH 7.4) for 5–10 min. The dissected thymus was postfixed in paraformaldehyde (4%) overnight at 4°C. To optically clear the thymus, we modified the ultimate 3D imaging of solvent-cleared organs (uDISCO) tissue clearing protocol by keeping the sample at 4°C for the entirety of the imaging session [55]. Postfixed thymus lobes were dehydrated with tert-butanol (Sigma-Aldrich; SHBM5332). Dehydration solutions were prepared by mixing tert-butanol and distilled water at various concentrations (30%/50%/70%/90%/100% tert-butanol). Next, the thymus was incubated in dichloromethane (DCM; Sigma-Aldrich, SHBJ8352) for the delipidation process. The tissue was then placed in BABB-D4, prepared by mixing BABB (benzyl alcohol + benzyl benzoate 1:2, Sigma-Aldrich; 24122 and W213802) with diphenyl ether (DPE; Alfa Aesar, A15791) at a ratio of 4:1 and adding 0.4% vol DL-alpha-tocopherol (Vitamin E; Alfa Aesar, A17039). Cleared thymus lobes were mounted in a custom-designed slide well filled with BABB-D4 and sealed with solvent-resistant silicone sealant (DOWSIL^™^ 730).

### Animal preparation for intravital thymus imaging

Mice were initially anesthetized (3–4% Isoflurane for induction, 1.5–2% for maintenance, 100% O_2_ at 1L/m) and then mounted to a heating pad to maintain normal body temperature. The right thoracic and abdominal regions were shaved, and the skin cleaned with 70% alcohol wipes. The mouse was then intubated using a laryngoscope and a 22-G angiocatheter (Exel Int, 26746). An injection of ketamine (100mg/kg) / xylazine (15mg/kg) was administered via IP injection before the thoracotomy. To perform the thoracotomy, an incision was made through the 2^nd^ intercostal space above the thymus and between the internal thoracic vein and sternum and expanded by inserting rib retractors into the intercostal space until the thymus was visible. A cauterizer was used to stop any excessive bleeding around the surgical site. The mice were euthanized after intravital imaging and the thymi were removed for *ex vivo* thymus imaging.

### Thymus holder placement

The adhesion stabilization holder consists of a stabilization ring with an inner diameter of 2.5 mm attached to an angled stabilization arm and was modeled in Openscad and 3D printed in polylactic acid (PLA). The holder was directly attached to the exposed thymus via a tissue safe adhesive (Vetbond, 084-1469SB) applied to the underside of the stabilization ring via a small paintbrush. After attachment, warm methyl cellulose (Sigma-Aldrich, M0512) was applied to the center of the holder to act as an immersion fluid for imaging.

### Two-photon microscopy

Imaging was performed with a custom-built two-photon video-rate microscope (Bliq Photonics) equipped with two femtosecond lasers (Insight X3 and MaiTai eHP DS, Spectra Physics). During intravital imaging, the Insight X3 and Maitai laser wavelengths were tuned to 800 nm and 950 nm, respectively, and *for ex vivo* imaging the Insight X3 and Maitai laser wavelengths were tuned to 820 nm and 1220 nm, respectively. Three fluorescent channels were acquired (503–538 nm, 572–608 nm, and 659–700 nm). For two-photon imaging, a 25x water immersion objective (Olympus; XLPLN25XWMP2) with a 1.05 numerical aperture was used to record video at 30/60/120 frames per second at a resolution of ~0.31 μm/pixel. To label thymus blood vessels *in vivo*, Evans’s blue was injected retro-orbitally after the thymus holder was placed.

### Image analysis

For image processing and measurements of blood vessel diameter, shear rate, leakage, frequency and area analysis, and manual blood flow quantification, Fiji (ImageJ 1.53f51) was used. All ImageJ scripts used for analysis and thymus adhesion holder 3D models are available online (https://github.com/SpencerLab-BIO/NativeThymusScripts). Image brightness/contrast was adjusted in Fiji for display purposes and images were cropped to remove vignetting. A previously published MATLAB (2020a) script was used to calculate blood flow automatically [56]. Cleared thymus datasets were stitched together using the Grid Stitching plugin in Fiji.

During intravital imaging, videos of blood flow in the thymus were recorded. Blood flow velocity was calculated with two methods. pLSPIV was used to calculate blood flow velocity automatically [56, 57]. However, when autmoatic blood flow measurements failed, blood flow velocity was calculated manually by tracking the change in the position of individual red blood cells (RBC) over time. Video frames were first aligned via the Linear Stack Alignment with SIFT plugin in Fiji and then the displacement of the approximate RBC centroid between frames was used to calculate blood flow per blood vessel.

Blood vessel diameters both *in vivo* and *ex vivo* were calculated via two different methods depending on the signal-to-noise ratio and the orientation of the blood vessel relative to the focal plane. When high contrast was observed between blood vessels and background, and vessels were orientated parallel to the focal plane, a modified version of the VasoMetrics2 [58] Fiji script was used. The only modifications made to the VasoMetrics2 script were done to improve user experience and the function related to vessel diameter calculation was not modified. Alternatively, when there was poor contrast between the vessels and background or vessels were orientated perpendicular to the focal plane, vessel diameter was measured manually via the Straight-line tool in Fiji. Blood vessel leakage measurements were taken at least 10 min after Evans blue injection by averaging 30 frames of blood vessel footage. Leakage was calculated as the ratio between average fluorescent intensity of a field of view (FOV) located at approximately the center of a blood vessel vs. a FOV immediately adjacent to the blood vessel. The shear rate for individual vessels was approximated as (8 × velocity) / diameter as previously described [59].

To measure blood vessel diameter, frequency, and area in the optically cleared thymus, 3D datasets of the thymus vasculature were downscaled by half, the Despeckle and Gaussian Blur (σ = 2) filters were applied, and the brightness/contrast was adjusted in Fiji. A mask of the outline of the thymus was then generated by downscaling the thymus by half and then either using a custom weka model generated by the Labkit plugin [60] or manually tracing the perimeter of the thymus. The mask of the thymus was then upscaled by 2 and a Euclidian Distance Map of the thymus was generated with the 3DSuite plugin [61]. The resulting distance map was thresholded to generate a mask corresponding to a volume ~150 μm from the edge of the thymus. To quantify blood vessel diameter and density in the cleared thymus, twenty random non-overlapping 300 x 300 μm FOV whose centroid was <150 μm from the edge of the thymus were used. The previously mentioned ~150 μm mask was used to limit the center of the FOV to within 150 μm from the edge of the thymus. Vessel diameter was calculated as previously described and vessel frequency was calculated as the number of vessels in the FOV divided by the area of the thymus in the FOV. The vessel area was calculated by thresholding the blood vessels in each FOV via the Otsu method and then calculating the % area in each FOV occupied by blood vessels.

To measure the thymus volume, the dissected thymus was placed on a gridded reference and the % area of the grid covered by the thymus was calculated [62].

### Statistical analysis

The Mann-Whitney U-test and Student’s t-tests were performed in GraphPad Prism to test for statistical significance, depending on whether datasets were normally distributed. A p-value < 0.05 was considered statistically significant (Data are presented as mean ± SD; *p<0.05, **p<0.01, ***p<0.001; ****p<0.0001).

## Results

### Experimental setup for intravital two-photon imaging of the native thymus

**Fig 1:**
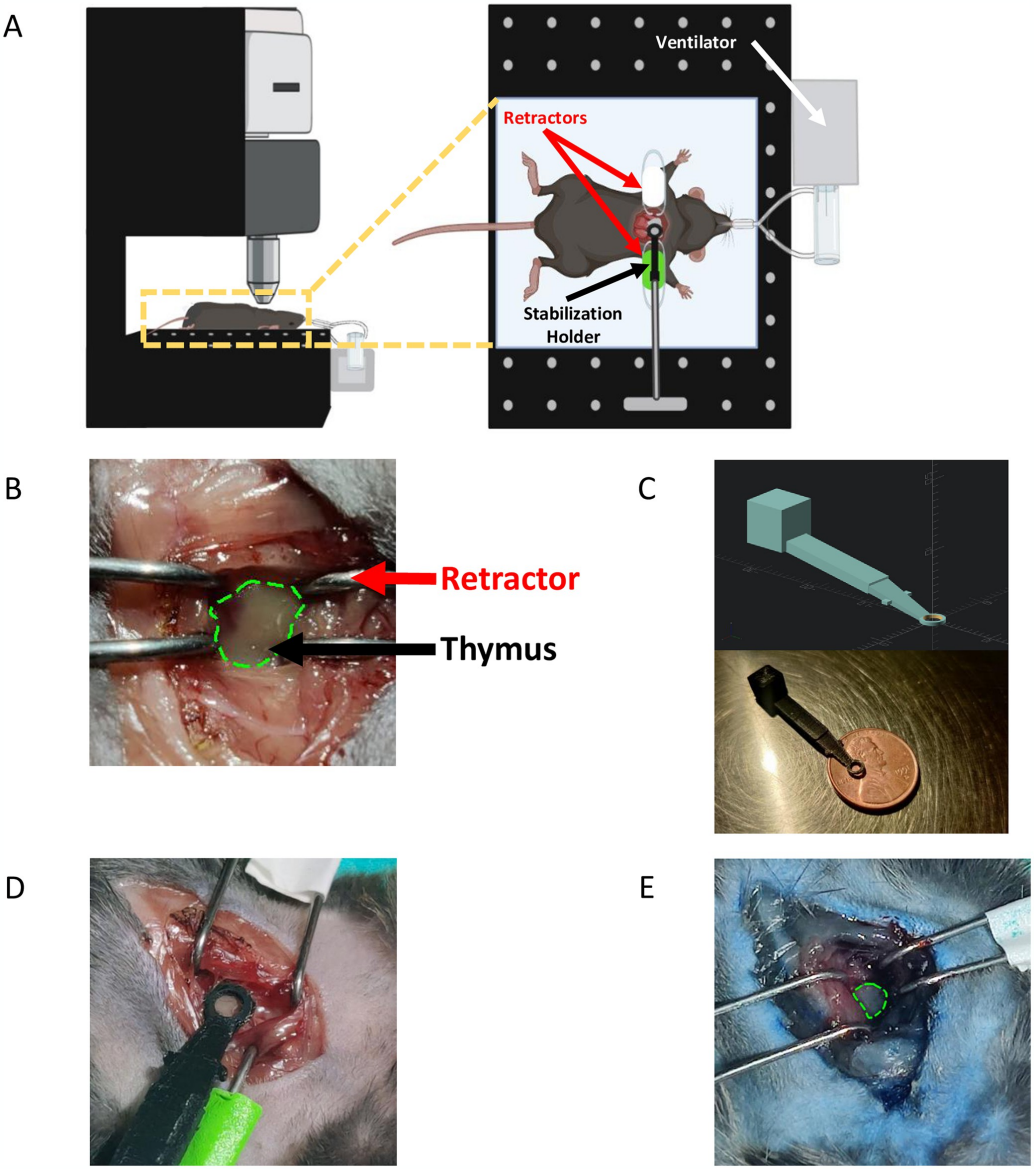
Experimental setup and thymus holder design. **(A)** Experimental schematic of intravital two-photon imaging of the native mouse thymus. **(B)** Image showing the exposed thymus through the 2^nd^ intercostal space after surgery. Green outline: thymus. **(C)** 3D model of thymus adhesion stabilization holder (top) and image of 3D-printed prototype (bottom). **(D)** Image showing adhesion stabilization holder attached to the exposed thymus after surgery. **(E)** Image showing the Evans blue perfused thymus after intravital imaging, indicating intact blood flow in the thymus at the time of injection. Green outline: thymus.

To image the native (i.e. *in situ*) murine thymus *in vivo*, we adapted methods for intravital cardiac microscopy [51, 54] to stabilize and visualize the thymus for intravital two-photon microscopy. Mice (8–12 weeks old) were anesthetized, ventilated, and placed in supine position on a temperature-controlled microscope stage (Fig 1A). Next, we performed a thoracotomy [48] through the second intercostal space to expose the thymus for imaging, and retractors were used to hold open the chest wall (Fig 1A and 1B). To stabilize the tissue during intravital imaging, we designed and 3D printed a custom thymus adhesion stabilization holder to reduce movement artifacts caused by cardiac and respiratory motion (Fig 1C). The stabilization holder consists of a flat ring attached to a support arm mounted to the microscope stage. The underside of the ring was covered with a thin layer of Vetbond tissue adhesive (3M Vetbond), which bonds to the underlying thymic tissue and provides a stable FOV for intravital two-photon microscopy (Fig 1D). Proper placement of the stabilization holder minimizes lateral and axial movement of the thymus preventing blurring and blinking artifacts during imaging (S1A and S1B Fig). Using this imaging setup, it was possible to have a clear imaging field within the thymus at a maximum diameter of 2.5 mm. To confirm thymic tissue and vascular viability during imaging, Evans blue dye was injected retro-orbitally and visible perfusion of the thymus was observed *in vivo* and *ex vivo* (Fig 1E and S1C Fig).

### *In vivo* imaging of the thymus

**Fig 2:**
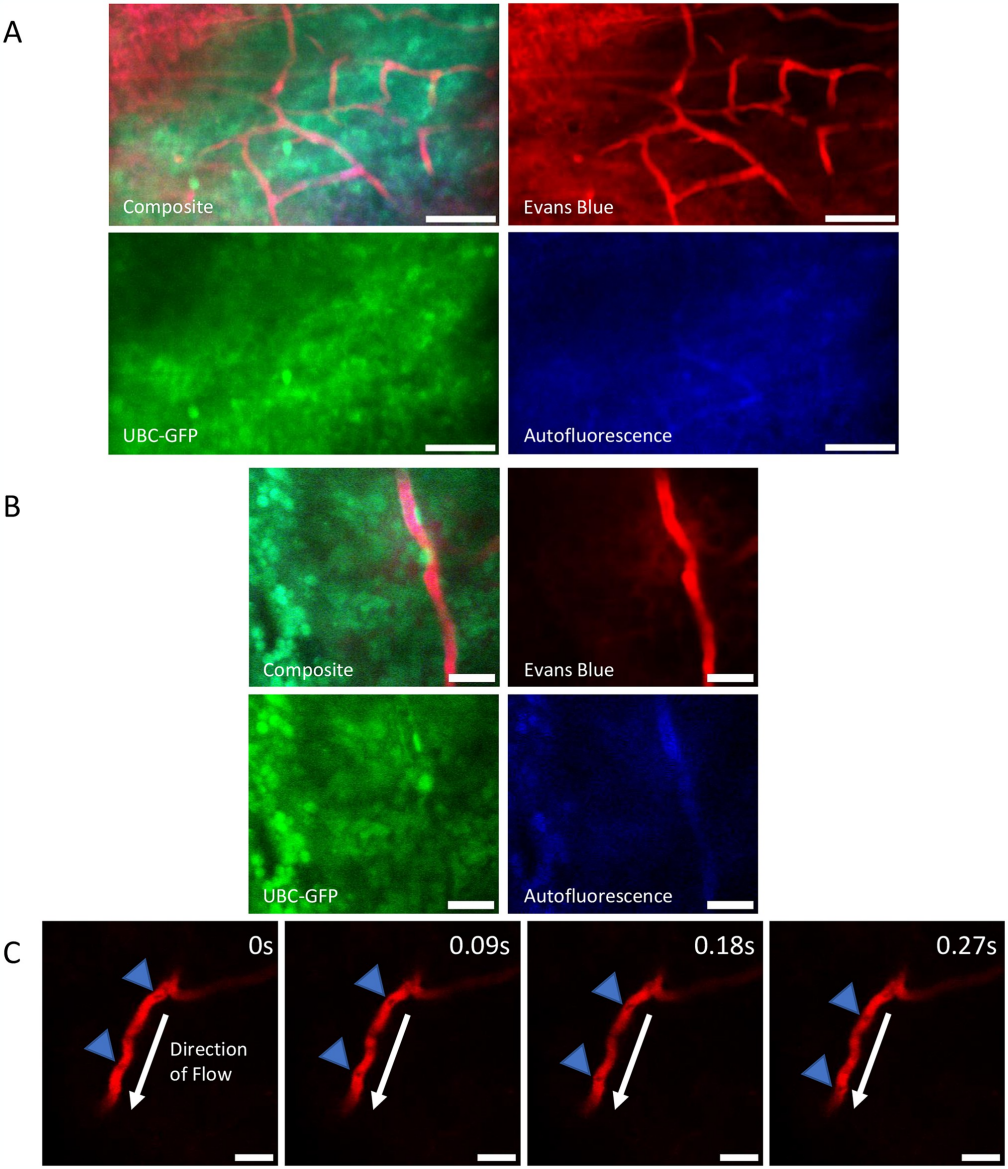
Intravital imaging of blood flow in the native thymus. **(A)** Representative maximum intensity projection of the untreated thymus from a UBC-GFP mouse *in vivo*. Red: blood vessels (Evans Blue)/thymus capsule; Green: GFP; Blue: autofluorescence. Scale bars ~ 50 μm. **(B)** Example average intensity projection of the untreated thymus from a UBC-GFP mouse demonstrating the ability to visualize individual GFP+ cells in the thymus *in vivo*. Red: blood vessels (Evans Blue); Green: GFP; Blue: autofluorescence. Scale bars ~ 25 μm. **(C)** Example negative contrast labeled blood flow in the untreated thymus from a UBC-GFP mouse showing the movement of RBCs through blood vessels over time. Red: blood vessels (Evans Blue); Blue Arrow: negative contrast labeled RBC; White Arrow: direction of blood flow. Scale bars ~ 25 μm.

We recorded two-photon images and videos in the native thymus of live mice using the methodology described above (Fig 2A–2C and S1–S3 Movies; n = 4 mice). Representative images and videos show the functional blood vessel network and individual GFP-labeled cells within UBC-GFP mice (Fig 2A–2C and S1–S3 Movies). UBC-GFP mice were used for imaging due to the universal GFP expression in all cells making the thymus easy to identify during two-photon imaging without requiring exogenous labels. In these representative images and movies of the thymus, we can clearly see the thymus capsule, cells that compose the thymus microenvironment, and the blood vessel network. The thymus capsule is visible in the upper left corner of Fig 2A due to non-specific labeling from Evans blue and confirmed by the lack of GFP+ cells and blood vessels. The stabilization adhesion holder minimized the motion generated by the heart and intubated lungs, allowing for optical sectioning and clear visualization of individual cells with subcelluar resolution (Fig 2B and S1B Fig). In addition, we confirmed the presence of blood flow within individual blood vessels in the thymus via the movement of RBCs revealed by negative contrast labeling (Fig 2C, S2 and S3 Movies). To confirm we imaged within the thymus, we imaged the excised thymus *ex vivo* with two-photon microscopy and detected very similar structures to the intravital images and movies (S2A Fig).

### Effects of irradiation on the thymus vasculature

**Fig 3:**
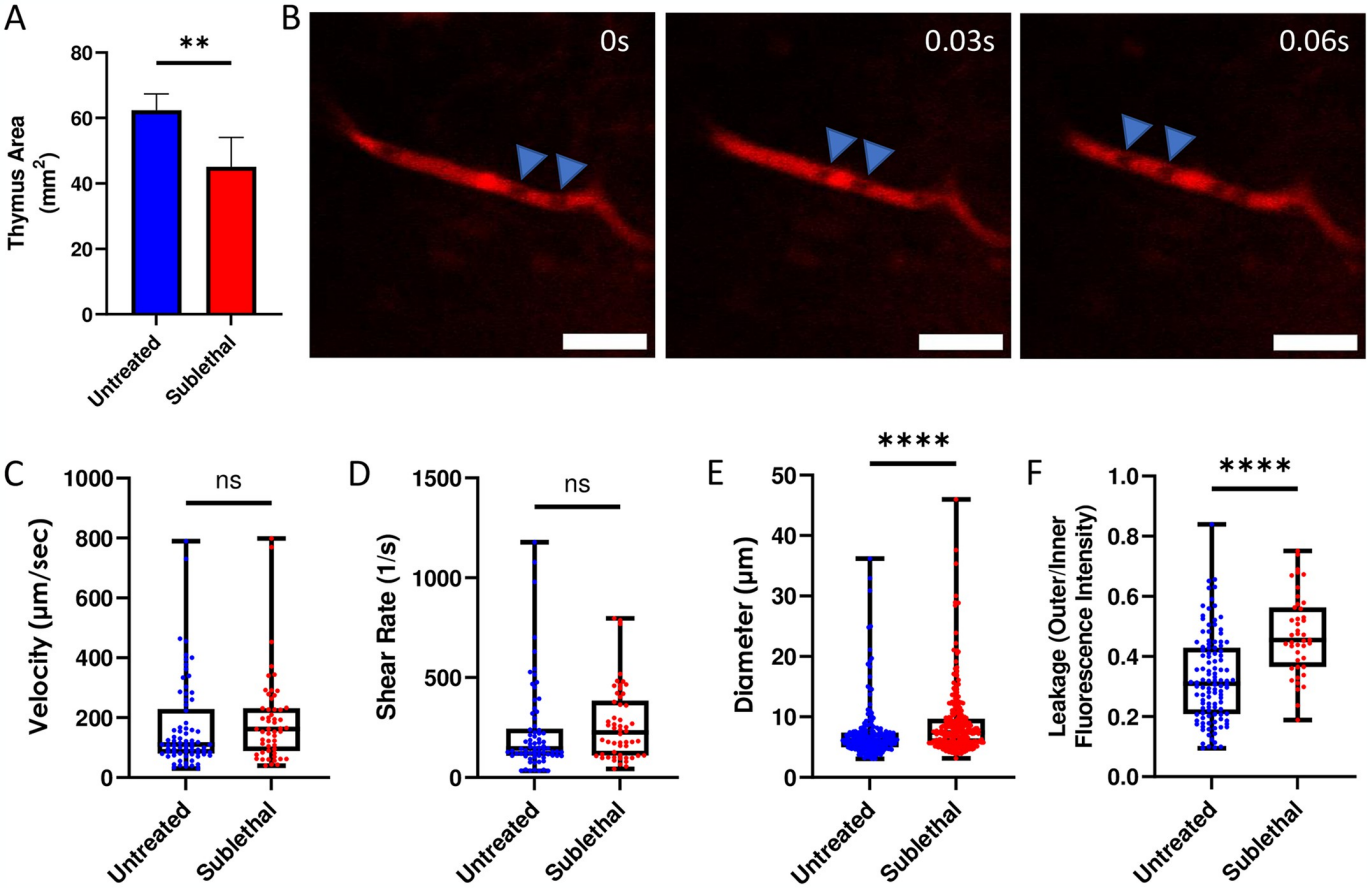
*In Vivo* comparison of native thymus vasculature in SL-TBI and untreated mice. **(A)** Quantification of thymus area from untreated and SL-TBI mice. **(B)** Example negative contrast labeled blood flow in the SL-TBI thymus from a UBC-GFP mouse showing the movement of RBCs through blood vessels over time. Red: blood vessels (Evans Blue); Blue Arrows: negative contrast labeled RBCs. Scale bars ~ 25 μm. Quantification of blood flow velocity **(C)**, vessel shear rate **(D)**, vessel diameter **(E)**, and vessel leakage **(F)** in the thymus of untreated and SL-TBI UBC-GFP mice.

Next, we hypothesized that changes to the blood vessel network structure from SL-TBI would alter hemodynamics and potentially barrier function in some microvessels of the cortex. To investigate this hypothesis, we performed SL-TBI (4.5 Gy) on UBC-GFP mice (n = 3 mice), imaged them 24 hours afterward, and compared them to age-matched untreated UBC-GFP controls that were not given SL-TBI. Despite a significant reduction (difference = 27.7%, p = 0.0043) in the size of the thymus after SL-TBI (Fig 3A and S2B Fig), we were able to confirm the presence of blood flow within individual vessels in the irradiated thymus (Fig 3B, S4 and S5 Movies). We observed a nonsignificant difference (p = 0.1244) in thymic blood flow velocity between SL-TBI treated and untreated mice (mean velocity = 191.8 μm/s and 169.2 μm/s, respectively; Fig 3C). In addition, a nonsignificant difference (p = 0.890) was observed in the shear rate between the SL-TBI treated and untreated mice (mean = 264.7 s^-1^ and 231.4 s^-1^, respectively; Fig 3D). Nevertheless, we observed a small but statistically significant increase (p < 0.0001) in mean blood vessel diameter of SL-TBI treated vs. untreated mice (mean blood vessel diameter = 8.9 μm and 7.3 μm, respectively; Fig 3E). Due to imaging depth limitations inherent to intravital two-photon microscopy, most vessels measured were likely capillaries within the thymus cortex. However, our observation of large diameter (>10 μm) blood vessels indicates that for some animals, it may be possible to image to the cortical-medullary boundary or even the medulla (Fig 3E). We also observed that the blood vessel barrier function was altered from SL-TBI resulting in significantly higher (p < 0.0001) vessel leakage in SL-TBI treated vs. untreated mice (mean leakage value = 0.4734 vs. 0.3278, respectively; Fig 3F). Following intravital imaging, thymi of SL-TBI mice were excised and imaged again *ex vivo*, revealing noticeable damage to the thymic microenvironment with widespread edema and an increase in autofluorescence (S2C Fig). Overall, using our novel thymic imaging method, we demonstrated that the vascular barrier is compromised as early as 24 hrs after SL-TBI even though the blood flow is largely unaltered at this time.

### *Ex vivo* imaging of cleared thymus tissue

**Fig 4:**
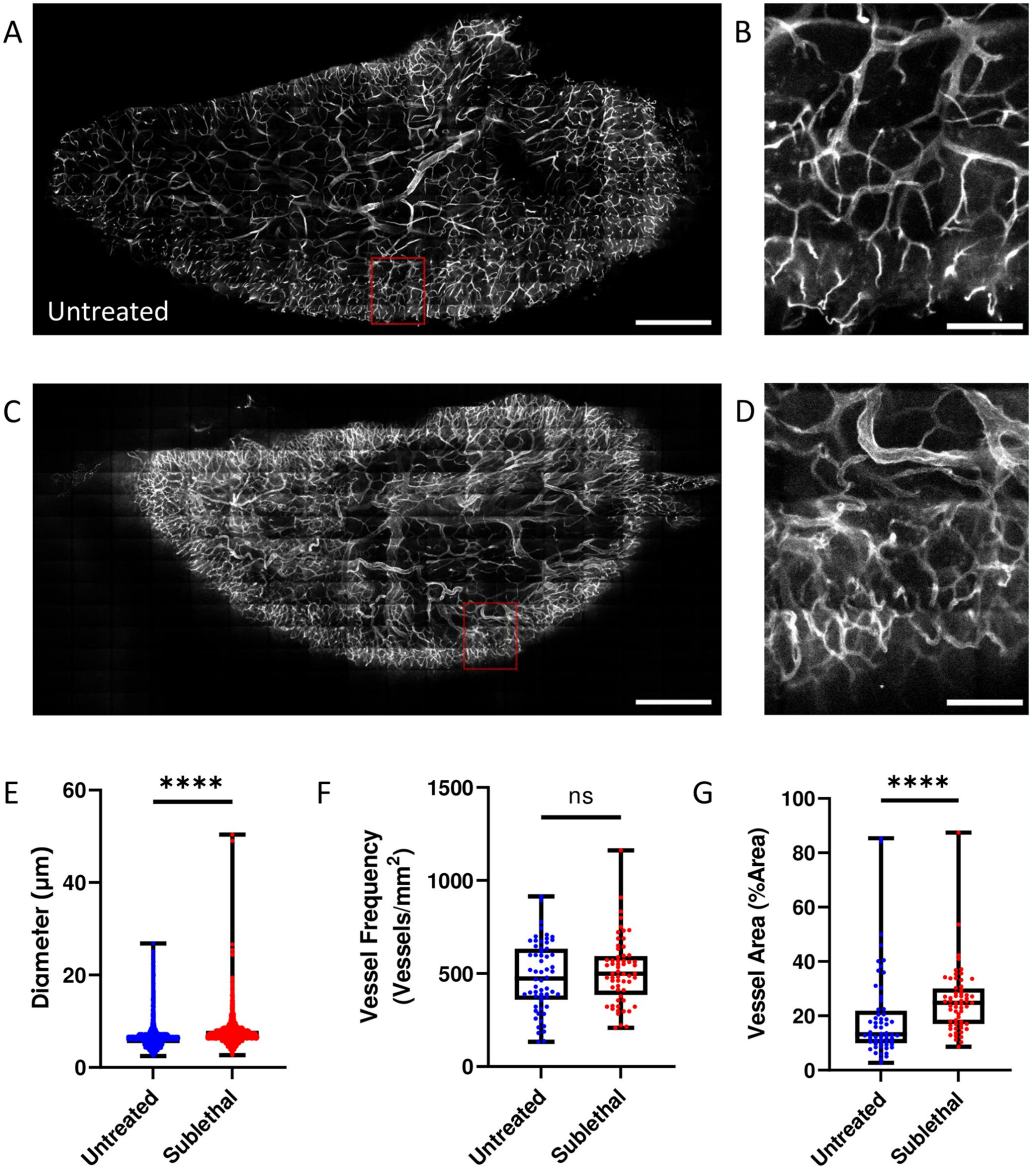
Changes to the thymus vasculature observed ex vivo. **(A)** Representative average intensity projection of the untreated thymus. Grey: blood vessels (labeled with Alexa647 conjugated antibodies against CD31, CD144, and Sca-1); Red Box: cropped FOV. Scale bar ~ 500 μm. **(B)** Cropped FOV of the untreated thymus. Grey: blood vessels (labeled with Alexa647 conjugated antibodies against CD31, CD144, and Sca-1). Scale bar ~ 100 μm. **(C)** Representative average intensity projection of the SL-TBI thymus. Grey: blood vessels (labeled with Alexa647 conjugated antibodies against CD31, CD144, and Sca-1); Red Box: cropped FOV. Scale bar ~ 500 μm. **(D)** Cropped FOV of the SL-TBI thymus. Grey: blood vessels (labeled with Alexa647 conjugated antibodies against CD31, CD144, and Sca-1). Scale bar ~ 100 μm. Quantification of blood vessel diameter **(E)**, blood vessel frequency **(F)** and blood vessel area **(G)** in the untreated and SL-TBI thymus ex vivo.

To further investigate anatomical changes to the native thymus blood vessel network, we imaged optically cleared whole thymus lobes (n = 3 mice each for SL-TBI treated and untreated controls) using a modified uDISCO protocol, where the sample is chilled for the entirety of the imaging session [55, 63]. Visualization of the whole cleared thymus using two-photon microscopy 24 hrs after SL-TBI revealed widespread damage to the blood vessel architecture when compared to the untreated mice (Fig 4A–4D, S6 and S7 Movies). To quantify these changes, we measured the blood vessel diameter in the cortex and found a significant increase in diameter in the SL-TBI treated vs. untreated mice (mean blood vessel diameter = 7.0 and 6.3 μm, respectively; p < 0.0001) confirming our *in vivo* findings (Fig 4E). We did not observe a significant difference (p = 0.3497) in thymus cortical vessel frequency (defined as the number of vessel segments per mm^2^) between SL-TBI treated and untreated mice (vessel frequency = 514.2 and 482.9 segments/mm^2^, respectively; Fig 4F). Further analysis revealed a significant increase (p < 0.0001) in thymus cortical blood vessel area (defined as the number of vessel pixels in a FOV over the total number of pixels in the image) between the SL-TBI treated and untreated mice (mean % vessel area = 25.5% vs. 19.3%, respectively; Fig 4G). Taken together, these results confirm that damage to the thymus vascular network from SL-TBI leads to an increase in vessel diameter and vessel volume after 24 hrs even though the overarching vascular network superstructure remains.

## Discussion

Studies of T cell development and thymus regeneration have typically relied on techniques such as flow cytometry, immunohistochemistry, transplantation [23, 27, 33, 35–40], and *ex vivo* imaging [30–32, 34]. While these techniques provide valuable information about thymus biology and T cell development, only direct intravital visualization of the native thymus enables accurate characterization of the spatiotemporal dynamics of blood flow while maintaining the natural chemical mileau. Therefore, we developed a novel intravital imaging technique to surgically access the thymus and directly visualize it with two-photon microscopy in live mice. We were able to record stable videos and images within the native thymus and measure blood flow velocity, blood vessel diameter, shear rate, and blood vessel leakage. Although the thymus medulla and/or cortex were not specifically labelled during imaging, we observed several larger blood vessels greater than 10 μm in diameter (up to 45 μm) deep within the thymus, indicating that our imaging method is potentially capable of visualizing the thymus medulla. This is consistent with the observation that the cortex is filled with primarily small capillaries where the medulla contains a variety of small and large blood vessels [64–67]. In addition, we report the ability to image as deep as 150 μm within the thymus, which would correspond to the cortico-medullary junction and/or medulla in some locations of the thymus. Further work will help to clarify this finding.

While alterations to the thymus vascular structure have been reported 4 days after SL-TBI [20], to our knowledge, no investigation has observed the effects of SL-TBI on native thymus hemodynamics. Using our imaging technique, we were able to study changes to hemodynamics in the thymus within 24 hours of SL-TBI. Although we observed a subtle but significant increase in blood vessel diameter and leakage shortly after SL-TBI, we found no significant difference in either blood flow velocity or shear rate. This data suggests that although the blood-thymus barrier has been compromised, as indicated by increased leakage, the blood flow and shear rate are largely unaffected at this timepoint. These findings raise several questions regarding the role hemodynamics and blood vessel integrity may play in promoting thymus regeneration, such as creating a more open blood-thymus barrier for ETP homing and transmigration, and the abnormal exposure of thymocytes and stromal cells to blood plasma constituents. Intravital study of the blood-thymus barrier at later time points would likely provide additional information about the role it plays in supporting ETP homing and cellular expansion, but thymus shrinkage [20] increases the difficulty of imaging at later timepoints after SL-TBI. Future technical innovations such as endoscopic imaging [68–70] and improved stabilization techniques [51, 54] may allow for the study of the thymus blood vascular system and microenvironment across the entire thymus recovery period.

In addition, we used a modified uDISCO tissue clearing method [55] combined with whole organ *ex vivo* two-photon imaging to validate the observed changes to the blood vessel network following SL-TBI. We characterized the thymic blood vascular system and quantified changes to the vessel diameter, volume, and frequency. Our imaging results revealed dramatic changes to the overall structure of the blood vessel network with a significant increase in vessel diameter and volume. Despite this, there were no significant differences in vessel frequency in SL-TBI thymi compared to the untreated control group. Coupled with the observation that the thymus undergoes significant shrinkage after SL-TBI, these results are in line with previous studies that showed the thymus vasculature is relatively radioresistant [20].

Overall, this study demonstrates a powerful new imaging method to directly investigate the native thymus in live mice and enables the characterization of the thymic microenvironment in ways not previously possible. We demonstrated that shortly after SL-TBI, the thymus vascular structure undergoes rapid and significant changes which may be of clinical relevance in the context of thymus regeneration and immune system recovery following HCT.

## Supporting information

S1 Fig Thymus stabilization via an adhesion holder. **(A)** Representative diagram of an unstabilized (left) and stabilized (right) thymus during imaging. Red: blood vessels/thymus capsule; Green: GFP; Black: adhesion holder. **(B)** Representative montages of blood vessels in well- and poorly-stabilized thymi. Red: blood vessels (Evans blue). Scale bars ~ 50 μm. **(C)** Representative images of dissected thymi from untreated (left, shown in Fig 1E) and SL-TBI (right) mice after receiving an Evans blue injection, demonstrating successful perfusion of the thymus.
(TIF)

S2 Fig Dissected and ex vivo thymus. **(A)** Representative average intensity projection of the thymus from an untreated mouse ex vivo. Red: blood vessels (Evans blue); Green: GFP; Blue: autofluorescence. Scale bars ~ 50 μm. **(B)** Representative size difference of thymi from untreated (left) and SL-TBI (right) mice. Black square border ~ 5 mm. **(C)** Representative average intensity projection of the thymus from a SL-TBI mouse ex vivo. Red: blood vessels (Evans blue); Green: GFP; Blue: autofluorescence. Scale bars ~ 50 μm.
(TIF)

S1 Movie Representative intravital two-photon zstack of the native thymus. Representative intravital two-photon zstack of the native thymus in a 10 week old UBC-GFP mouse. Red: blood vessels (Evans blue); Green = GFP; Blue = autofluorescence. Scale bar ~ 50 μm; Zstep size = 2 μm.
(AVI)

S2 Movie Representative blood flow within the untreated native thymus. Representative video showing blood flow of the thymus in vivo in an untreated 10 week old UBC-GFP mouse. Red: blood vessels (Evans blue); Green = GFP; Blue = autofluorescence. Scale bar ~ 50 μm.
(AVI)

S3 Movie Representative blood flow within the untreated native thymus. Red channel only video corresponding to S2 Movie. Grey: blood vessels (Evans blue). Scale bar ~ 50 μm.
(AVI)

S4 Movie Representative blood flow within the SL-TBI native thymus. Representative video showing blood flow of the thymus in vivo in a SL-TBI 10 week old UBC-GFP mouse. Red: blood vessels (Evans blue); Green = GFP; Blue = autofluorescence. Scale bar ~ 50 μm.
(AVI)

S5 Movie Representative blood flow within the SL-TBI native thymus. Red channel only video corresponding to S4 Movie . Grey: blood vessels (Evans blue). Scale bar ~ 50 μm.
(AVI)

S6 Movie 3D Model of the optically cleared thymus vasculature. Representative 3D model of the optically cleared thymus vasculature from an untreated mouse. Grey = blood vessels (labeled with Alexa647 conjugated antibodies against CD31, CD144, and Sca-1). Scale bar ~ 250 μm.
(AVI)

S7 Movie 3D Model of the optically cleared thymus vasculature. Representative 3D model of the optically cleared thymus vasculature from a SL-TBI mouse. Grey = blood vessels (labeled with Alexa647 conjugated antibodies against CD31, CD144, and Sca-1). Scale bar ~ 250 μm.
(AVI)
