# Supplementary figures and images for: Intravital two-photon microscopy of the native mouse thymus
Source: PLoS One. 2024 Aug 1;19(8):e0307962. doi: 10.1371/journal.pone.0307962 (PMC11293686; doi:10.1371/journal.pone.0307962)

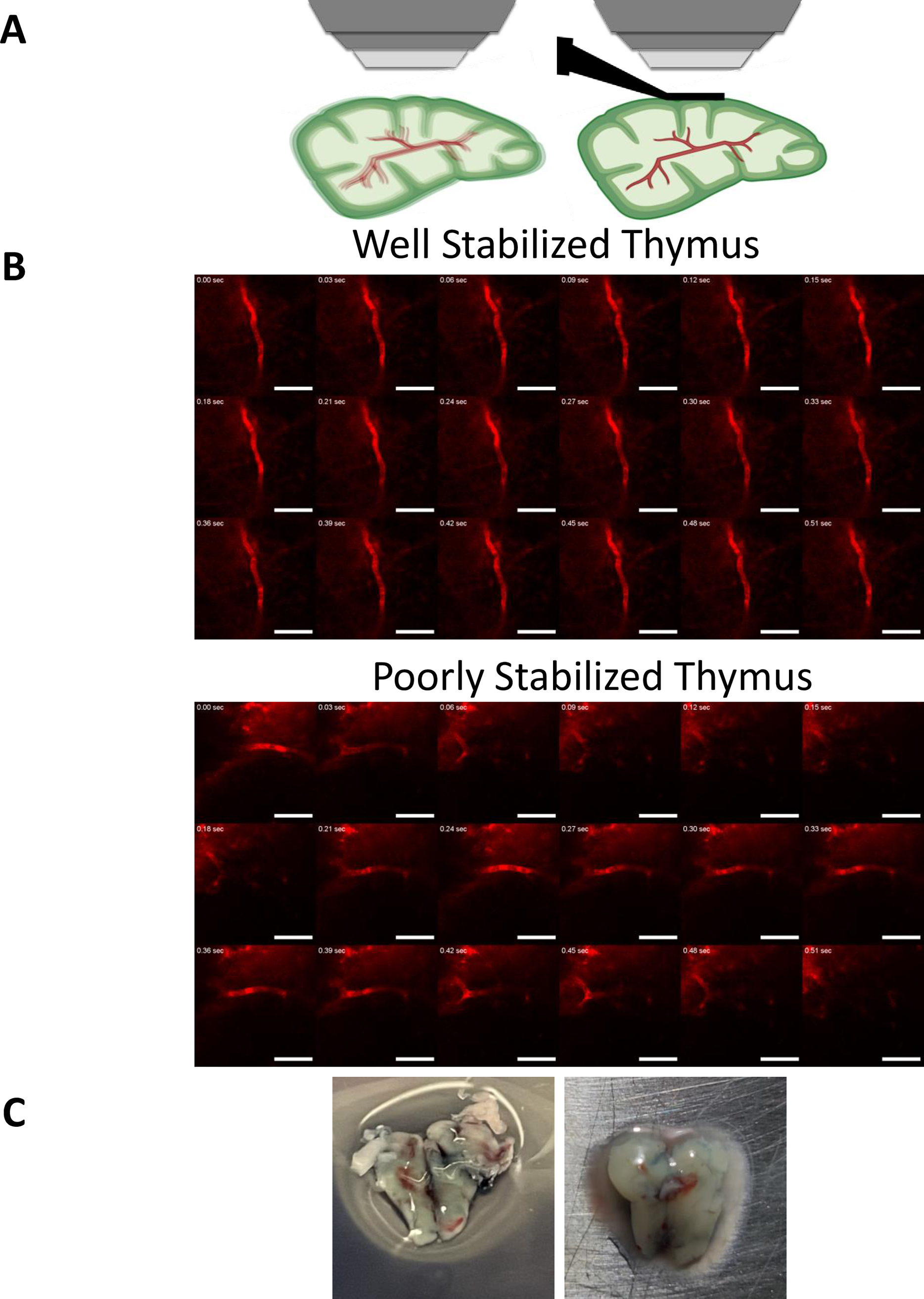

Supplement: S1 Fig — (A) Representative diagram of an unstabilized (left) and stabilized (right) thymus during imaging. Red: blood vessels/thymus capsule; Green: GFP; Black: adhesion holder. (B) Representative montages of blood vessels in well- and poorly-stabilized thymi. Red: blood vessels (Evans blue). Scale bars ~ 50 μm. (C) Representative images of dissected thymi from untreated (left, shown in Fig 1E) and SL-TBI (right) mice after receiving an Evans blue injection, demonstrating successful perfusion of the thymus. (TIF) [file pone.0307962.s001.tif]

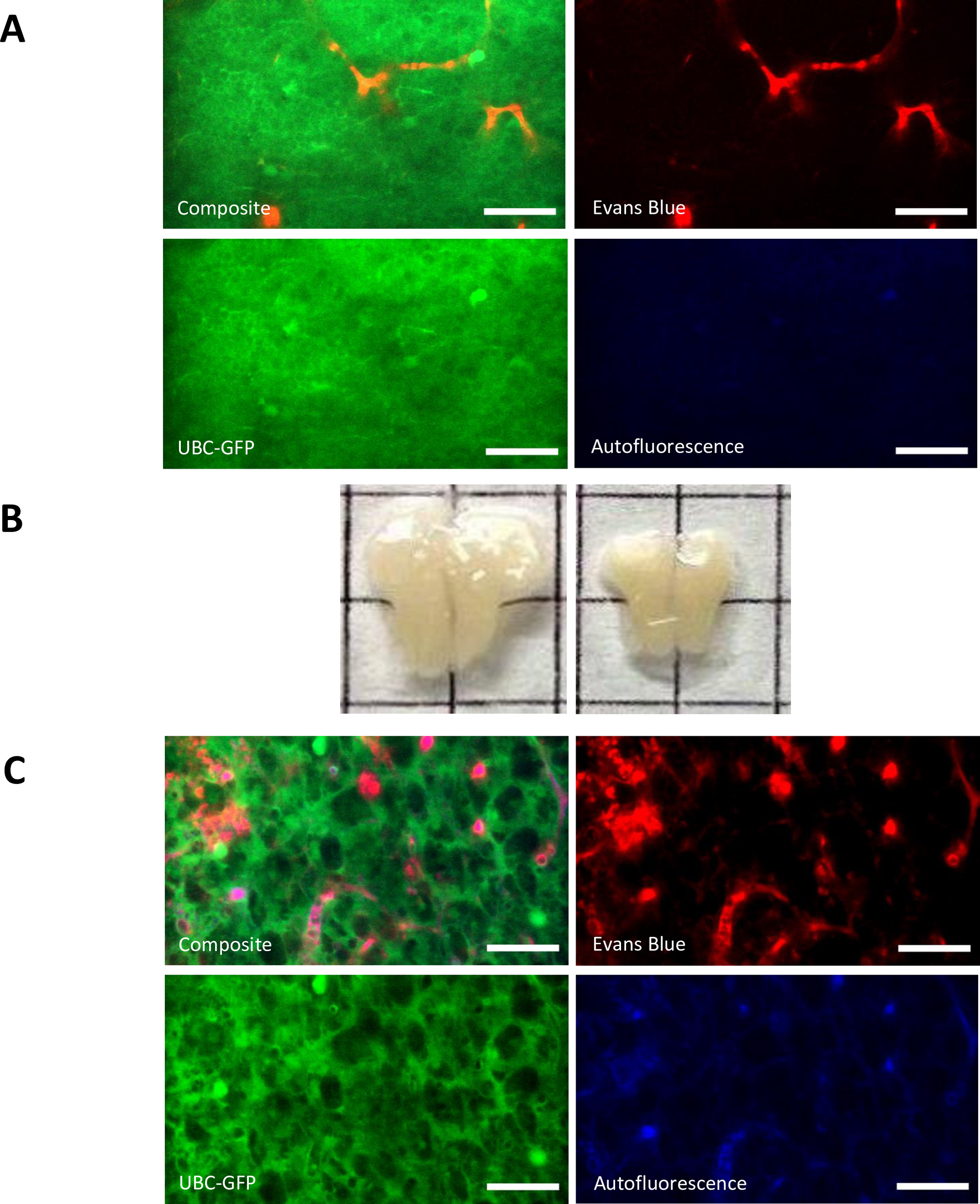

Supplement: S2 Fig — (A) Representative average intensity projection of the thymus from an untreated mouse ex vivo. Red: blood vessels (Evans blue); Green: GFP; Blue: autofluorescence. Scale bars ~ 50 μm. (B) Representative size difference of thymi from untreated (left) and SL-TBI (right) mice. Black square border ~ 5 mm. (C) Representative average intensity projection of the thymus from a SL-TBI mouse ex vivo. Red: blood vessels (Evans blue); Green: GFP; Blue: autofluorescence. Scale bars ~ 50 μm. (TIF) [file pone.0307962.s002.tif]
